# Supplementary figures and images for: Co-existence of synaptic plasticity and metastable dynamics in a spiking model of cortical circuits
Source: PLoS Comput Biol. 2024 Jul 1;20(7):e1012220. doi: 10.1371/journal.pcbi.1012220 (PMC11244818; doi:10.1371/journal.pcbi.1012220)

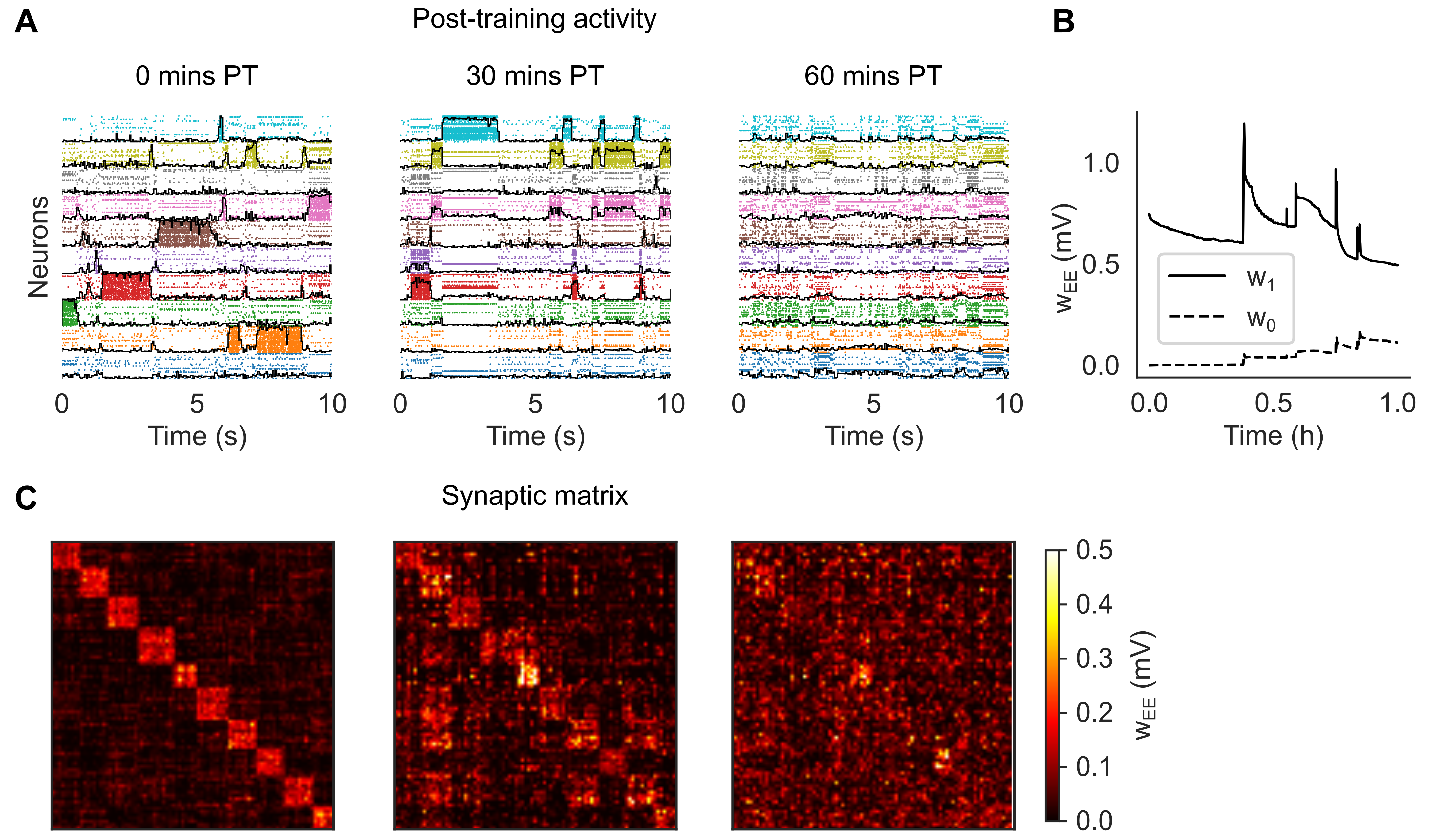

Supplement: S1 Fig — A: Rasterplot of excitatory neurons taken immediately after training (left), 30 minutes after training (middle), and 60 minutes after training (right). Same keys as Fig 2 of the main text. B: Averaged post-training excitatory synaptic weights as a function of time. w1: mean weights across synapses connecting neurons sharing at least one stimulus; w0: mean weights across synapses connecting neurons sharing no stimuli. C: Synaptic matrix of the network at the same times as in A showing the formation of clusters from the block structure of the matrix. (TIFF) [file pcbi.1012220.s001.tiff]

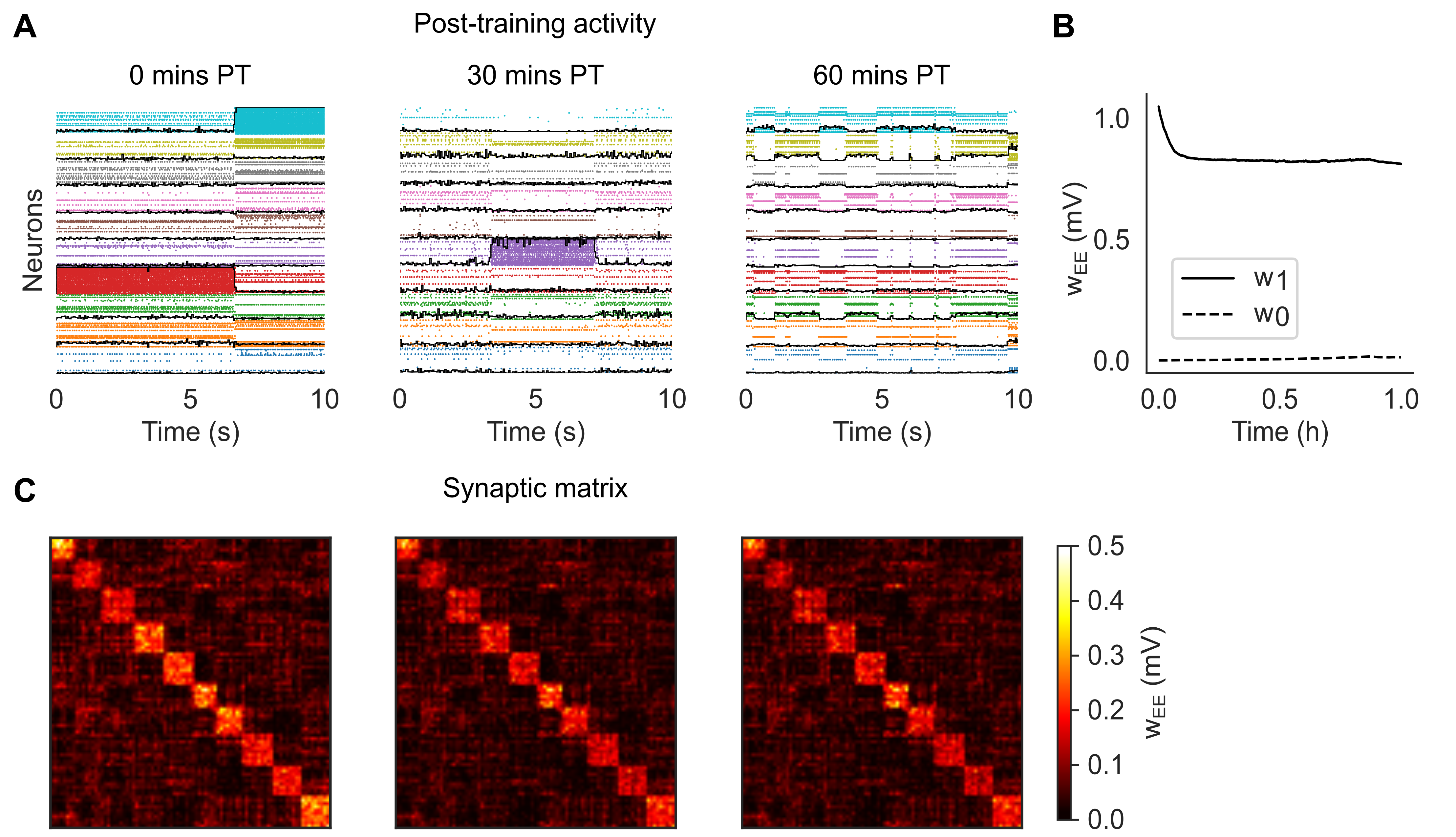

Supplement: S2 Fig — Same keys as S1 Fig. (TIFF) [file pcbi.1012220.s002.tiff]

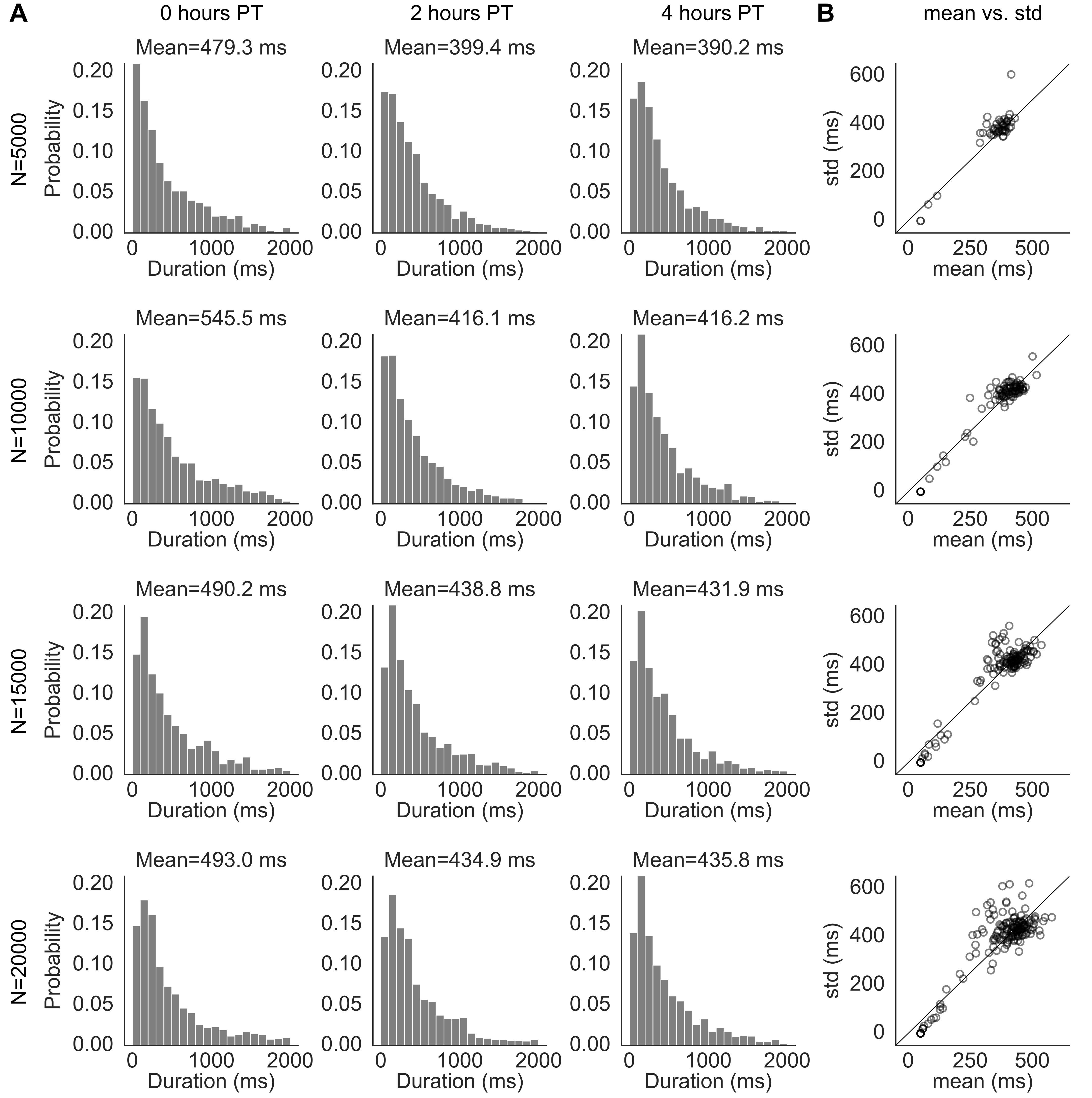

Supplement: S3 Fig — A: Distributions of durations right after training (left column), 2 hours post-training (middle) and 4 hours post-training (right) for different network sizes. Means tend to decrease with post-training time and increase with network size, approaching stability 4 hours post-training and for N ≥ 15, 000. B: Scatterplots of standard deviation vs. mean of durations for the corresponding networks in A, superimposed to the identity line. Each circle corresponds to a cluster. For the majority of the clusters, the standard deviations are approximately equal to the means as expected for an exponential distribution. (TIFF) [file pcbi.1012220.s003.tiff]

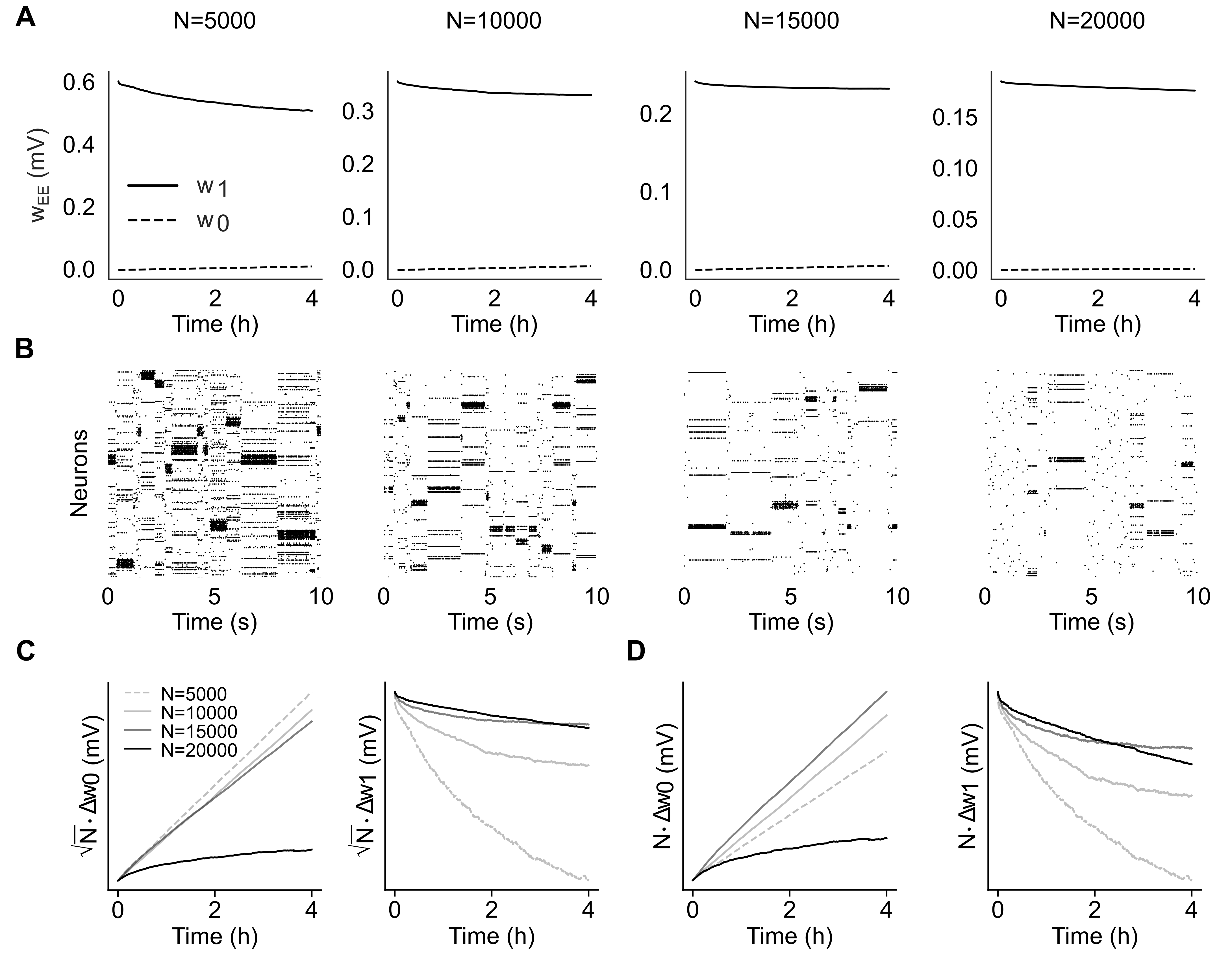

Supplement: S4 Fig — Same as Fig 4 of the main text but for a different scaling, namely f∝1/N,Q∝N (see the main text). A: Time dependence of average synaptic weights w1 and w0 after training for networks of different size N, with NE = 0.8N excitatory neurons. Scaling laws were f = 1/Q with Q=N/10, giving NQ=fNE=0.810N neurons in each cluster (where Q and NQ were rounded to the nearest integer). From left to right, Q = 22, 32, 39 and 45. B: Raster plots of the network’s activity 4 hours after training for the corresponding networks in A. C: Plots of NΔw0 and NΔw1 vs. time after training for the different network in A (note the difference with of Fig 4C of the main text). Observations were taken 0 to 4 hours post training. D: Same as C for NΔwC vs. time. (TIFF) [file pcbi.1012220.s004.tiff]
